# Pharmacists’ Perception of the Coronavirus Pandemic (COVID-19) in Jordan: A Cross-Sectional Study

**DOI:** 10.3390/ijerph182111541

**Published:** 2021-11-03

**Authors:** Tamara Al-Daghastani, Odate Tadros, Shereen Arabiyat, Deema Jaber, Husam AlSalamat

**Affiliations:** 1 Department of Medical Allied Sciences, Al-Balqa Applied University, Al-Salt 19117, Jordan; o.tadros@bau.edu.jo (O.T.); Shereen.arabiyat@bau.edu.jo (S.A.); 2 Biopharmaceutics and Clinical Pharmacy Department, School of Pharmacy, Zarqa University, Amman 11942, Jordan; dsuleiman@zu.edu.jo; 3 Department of Basic Medical Sciences, Faculty of Medicine, Al-Balqa Applied University, Al-Salt 19117, Jordan; h.alsalamat@bau.edu.jo; 4 Department of Biopharmaceutics and Clinical Pharmacy, School of Pharmacy, The University of Jordan, Amman 11942, Jordan

**Keywords:** COVID-19, Jordan, perception, pharmacists

## Abstract

Objectives: To analyze the role of pharmacists during the COVID-19 pandemic, to measure pharmacists’ attitude toward COVID-19 safety measures (wearing masks, wearing gloves, isolation shield, good hygiene, etc.), and explore their perspectives regarding a second wave of the virus. Methods: This cross-sectional online survey study was conducted in Jordan during the COVID-19 outbreak in July 2020 to discuss Jordanian pharmacists’ awareness of safety at their workplace during the COVID-19 outbreak, their sources of information, and their predictions for COVID-19 vaccination. Results: The participants (*n* = 311) were all pharmacists mostly aged between 23–30 years old (45%) and female (83%). The primary source of information about COVID-19 was social networking (38.9%). Pharmacists were committed to social distancing (86.5%) and wearing masks (76.2%). They expressed levels of agreement to their role in decreasing COVID-19 spread (94.2%) and correcting false information (94.5%); they expressed levels of expectation toward concern about a second COVID-19 wave (83%) that would be more severe than the previous one (43.4%). Pharmacists expected that an influenza vaccine might be helpful in decreasing severity and spread of the COVID-19 pandemic (56.9%). Pharmacists expected COVID-19 vaccine development within 6 months of administering our study survey (84.9%) and that vaccination might be effective in preventing COVID-19 (93%) infection. Conclusion: Pharmacists expressed positive roles on COVID-19 spread through exemplary actions, self-commitment to protection measures, and public health awareness. Social media as a source of health information should be cautiously investigated, and pharmacists should always refer to evidence-based sources. The role of pharmacists is particularly important for the upcoming era of COVID-19 vaccination administration and awareness.

## 1. Introduction

Coronavirus (CoV) infections are caused by emerging respiratory viruses and are known to cause illness ranging from common cold-like symptoms to severe acute respiratory syndrome (SARS) [1]. CoV are zoonotic pathogens that can be transmitted animal-to-human and human-to-human [2]. Since the emergence of the 2019 novel coronavirus (2019-nCoV) infection in Wuhan, China, in December 2019 [3], it spread rapidly across China and many other countries [2]. The World Health Organization (WHO) created a new name for the 2019-nCoV epidemic: coronavirus disease (COVID-19), on 11 February 2020 [4]. Subsequently, the Study Group of the International Committee on Taxonomy of Viruses identified this emerging virus as SARS-CoV-2 [4].

Fake news and rumormongering are popular in a wired world because of the increase in internet and social media use, especially during a pandemic [5]. A confused understanding of an emerging communicable disease, of which even the experts have insufficient knowledge, can lead to fear and excessive panic, which is likely to exacerbate the disease epidemic [6]. Similar phenomena emerged during the 2002–2004 SARS epidemic, and in America during the 2014 Ebola outbreak [7,8]. Such observations underline the critical task of communicating with the general public and healthcare professionals, and the value of managing their understanding of outbreak management of disease, which can impact community compliance with precautionary strategies. Understanding relevant factors affecting and motivating individuals to participate in precautionary actions can also assist decision-makers in taking effective action in order to improve individual or community health. It is therefore important to consider people’s perception of risk and to recognize their reliable sources of knowledge in order to interact effectively and frame key messages in response to the emerging disease [9].

Pharmacists, as healthcare providers, could be the first point of contact for many people with the healthcare system to discuss their health problems or when they simply need information. In these circumstances, the pharmacists’ function is not limited solely to ensuring sufficient stocks of medicines and protective equipment, but also to play an important role in minimizing transmission to the population by increasing public knowledge [10,11]. Based on the hypothesis that pharmacists carry a crucial role in shaping healthcare response to pandemics like COVID-19, we expect pharmacists to be equipped with sufficient knowledge of public health related information, express positive attitudes toward their role as healthcare providers, and commit to safety measures during pandemics. Accordingly, this study used social networks as a primary source with aims to analyze the role of pharmacists during the COVID-19 pandemic and to measure their attitudes toward COVID-19 safety measures, and their perspectives about a second wave of the virus. Additional knowledge aspects were challenged to assess pharmacist’s health literacy related to COVID-19, and their willingness to participate as COVID-19 vaccine providers.

## 2. Methods

### 2.1. Study Design

This is a descriptive, cross-sectional online study using a self-reporting survey distributed during July 2020 designed through Google forms platform. The survey was administered in Arabic, the official language of Jordan. A list of pharmacists was provided by the Jordan Pharmacists Association (JPA), which is the official and only syndicate for pharmacists in Jordan, established in 1957 with obligatory membership for pharmacists to gain their license to practice pharmacy in Jordan. The JPA has around 15,000 pharmacist members across Jordan. Considering the absence of similar measures of our study variables in Jordan, the response distribution was assumed as 75% and a sample size of at least 283 pharmacists was expected to express 95% confidence level with 5% error using the Raosoft sample size calculator.

### 2.2. Data Collection

Social media (mainly WhatsApp and Facebook) was used to spread the survey to reach pharmacists all over Jordan through groups that only included pharmacists. Close-ended questions were designed to measure our goals using predefined answers. The research proposal and tools were reviewed and approved by the Institutional Review Board (IRB) at Al-Balqa Applied University, Al-Salt, Jordan (code: 492/1/3/26). Participation in the study was completely voluntary and posed no risk upon participants. Completion of the study survey was deemed as an informed consent for participation in the study. Eligibility to participate included that participants were a pharmacist, while those who did not consent to participate or did not complete the questionnaire were excluded.

Validation of this study’s survey items for comprehension was carried out prior to launch using a random pilot sample of 20 independent pharmacy professionals, mainly serving as community pharmacists. Feedback regarding the clarity and comprehension of the pilot survey were conveyed to the research team and discussed thoroughly, then incorporated where appropriate produce a final version of the survey. All items of our study survey were based on current literature [12,13,14], and carefully discussed among our team of experts comprised of academics and clinical pharmacists.

Demographic data of participants includes: their residence area, gender, age, level of education, and place of work. We also asked the participants about their primary source of information during the pandemic.

### 2.3. Questionnaire Tool

Our survey included questions to measure the pharmacist’s opinion on their role during the COVID-19 pandemic. The study survey included questions to measure pharmacist’s commitment to follow protective procedures and the use of protective equipment as per regulations of the pandemic, including social distancing, wearing masks, and wearing gloves. Moreover, several questions seek to assess pharmacists’ perceptions toward their role in decreasing COVID-19 spread and correcting false information about the COVID-19 pandemic. Additionally, this survey challenges pharmacist’s knowledge by assessing their ability to differentiate between COVID-19, influenza, and Spanish influenza. A question was also included to measure perception of pharmacists’ ability to collect samples for COVID-19 testing.

Pharmacists’ expectations related to current and future COVID-19 pandemics were also assessed. Pharmacists were asked for their expectation of a second COVID-19 pandemic wave in terms of presence, relative severity to the first wave, and timing. Our survey also including pharmacists’ expectations about a COVID-19 vaccine, in terms of development and effectiveness in resolving the ongoing pandemic. Their expectation of whether immunization against influenza might decrease severity and the spread of COVID-19 was also assessed from the perspective of participating pharmacists in our study.

### 2.4. Statistical Analysis

All data were generated automatically to Microsoft Excel by Google forms then coded and analyzed using Statistical Package for Social Sciences (IBM Corp. Released 2013. IBM SPSS Statistics for Windows, Version 22.0. Armonk, NY, USA: IBM Corp.). Descriptive statistics with corresponding 95% confidence intervals (CIs) were constructed. Differences between various categorical variables were evaluated using the chi-square test.

## 3. Results

Data were collected from 311 participants, all pharmacists. A total of 45.0% of participants in this study were aged between 23–30 years old, 33.1% were aged between 31–40 years old, 15.8% were aged between 41–50 years old, and 6.1% were aged above 50 years old. Around 83.0% of the sample were females. In terms of education, 77.2% were university graduates with a bachelor’s degree, 17.7% with a master’s degree, and 5.1% held a doctoral degree. For residence location, most of the participants (38.3%) were in Amman, the capital city, while the rest of participants were distributed as living in the middle area of Jordan(35.0%), living in northern area (14.8%), and living in the southern area (11.9%). Among participants, most reported social networks as their primary source of information about the COVID-19 pandemic (38.9%), while other sources reported were TV newscasts (23.8%), specialized scientific journals (18.6%), personal readings (15.4%), and other sources (3.2%). Pharmacists in our study were mostly community pharmacists (39.5%), while 5.8% were hospital pharmacists, and 54.0% had work elsewhere to the defined place of work, Table 1.

Participating pharmacists in our study expressed commitment at all times to social distancing measures (86.5%), while 10.9% reported occasional commitment; only 1.3% self-reported non-commitment. Moreover, most participants, 76.2%, reported commitment at all times to wearing masks, while 16.7% reported occasional commitment, and only 6.4% reported non-commitment. Regarding their commitment to wearing gloves, most of our study participants reported commitment at all times (58.8%), while 28.9% reported occasional commitment, and only 12.2% reported non-commitment.

Most participants (48.9%) confirmed that pharmacists do have a role in decreasing the COVID-19 pandemic, while 45.0% believed they might have such a role, and only 5.8% denied having such a role. Participants mostly believed that pharmacists knew the difference between influenza and COVID-19 (84.6%), while 10.6% believed they might know, and only 4.5% denied such beliefs. Additionally, participants mostly believed that pharmacists might know the difference between the Spanish influenza of 1918 and the COVID-19 pandemic (33.4%), while 29.3% had never heard of the 1918 pandemic, 24.4% believed they did know the difference, and only 12.5% denied such belief of pharmacist’s knowledge. Most participants reported that pharmacists correct false information about the COVID-19 pandemic (76.2%), while 18.3% believed they might have such a role, and only 5.5% denied this role. Regarding pharmacist’s ability to take samples for COVID-19 testing, most participants believed that pharmacists are not competent for such a role (54.7%), while 23.8% believed they might be for such a role, and only 21.5% believed they do have such an ability.

Regarding their expectation of a second COVID-19 wave, most participants confirmed such expectation (48.6%), while 33.8% believed this might happen, and only 17.0% denied a second wave. Moreover, most participants expected that a second wave of COVID-19 would be more severe compared to the first one (43.4%), while 35.0% expected it to be less severe, 12.2% expected there was no relation of severity between both waves, and 8.7% expected the second wave to be of similar severity to the first one. Additionally, 58.5% of participants expected the second wave to happen within 3 months, while 23.2% expected it within one year, 3.2% expected it within two years, and 12.9% expected it after more than two years, Table 2.

A total of 56.9% of participants expressed varying levels of agreement toward the ability of an influenza vaccine to decrease the severity and spread of COVID-19: (26.4% clearly agree, and 30.5% might agree), although 43.1% clearly denied such ability of an influenza vaccine. Regarding expectation of developing a COVID-19 vaccine within 6 months of administering our study survey, 55.6% of participants believed this is expected, while 29.3% might expect this, and only 14.8% denied such expectation. Moreover, the efficacy of a COVID-19 vaccine was mostly expected by our study participants (60.8%), while 32.2% believed this might be expected, and only 7.1% denied its effectiveness, Table 3.

Factorial associations were established between several demographic characteristics of participants with other variables in our study. Pharmacists in hospital settings were more committed toward wearing gloves (*p*-value = 0.013). Regarding pharmacists’ role in decreasing COVID-19 spread, several participant segments expressed more significant involvement for such a role, including male participants (*p*-value = 0.010), participants living in the middle area of Jordan (*p*-value = 0.008), and hospital pharmacists (0.048). Female pharmacists significantly believed more of their knowledge of the difference between influenza and the COVID-19 pandemic (*p*-value = 0.032). Pharmacist knowledge of similarities between the COVID-19 pandemic and the Spanish influenza of 1918 expressed varying significant uncertainties, as pharmacists in the 23–30 age group (*p*-value < 0.001), and pharmacists with bachelor’s degree (*p*-value = 0.018) were both more uncertain of such knowledge.

Expectation of a second COVID-19 wave was more significantly noted by pharmacists living in the middle area of Jordan (*p*-value = 0.017). Participants holding master’s or doctoral degrees significantly expected a more severe second COVID-19 wave (*p*-value < 0.001) and they also significantly expected the occurrence of a second wave within three months (*p*-value = 0.016). Low expectation was significantly noted by pharmacists living in the capital (*p*-value < 0.001) regarding the ability of an influenza vaccine to decrease the severity and spread of COVID-19. Lastly, pharmacists in the 23–30 years old age group (*p*-value < 0.001) significantly believed that a COVID-19 vaccine would be effective in COVID-19 prevention, Table 4.

## 4. Discussion

In this cross-sectional study, attempts have been made to discuss the perception of pharmacists in Jordan during the coronavirus pandemic (COVID-19) using an online survey. Our study uncovered several roles of pharmacists related to their knowledge, attitudes, and practices during the COVID-19 pandemic. These included pharmacists’ commitment to protective measures and health education, which both honor the pharmacist’s role of leading by example and spreading evidence based scientific information regarding this newly emerging virus, ultimately altering community response to pandemics such as COVID-19. Additionally, this study assessed several expectations of pharmacists about current and future COVID-19 pandemics.

Healthcare staff, including pharmacists, have a reported increased risk of positive COVID-19 testing [15]. This sheds light for several studies to discuss the obligations to provide necessary protective equipment for healthcare workers in order to fight emerging diseases [16]. The majority of pharmacists in our study were committed to protective measures for COVID-19, including: social distancing, wearing masks, and wearing gloves. Not surprisingly, in our study, pharmacists working in hospitals were more committed to wearing protective clothing like gloves, making them more considerate of their established increased risk of getting infected. Pharmacists in other settings should take similar rigorous self-protective measures, because of the reported lack of commitment by patients in different settings [17].

Pharmacists are pillars of a more safe and effective healthcare service. In our study, pharmacists believed they had an influential role in decreasing COVID-19 spread, and the role of hospital pharmacists was particularly prevalent. Our findings are consistent with a previous independent study completed by Mukattash. T. et al., which revealed a positive role of Jordanian pharmacists during the COVID-19 pandemic, particularly ones related to patient safety and satisfaction [18]. Hussain A. et al. attributed this role of pharmacists to the quick establishment of protective guidance for pharmacists, contributions to drug formularies, addressing drug shortages, providing public health education for management and prevention of infection, contributing in clinical trials, and drug evaluation [19].

Pharmacists are probably the most accessible members of a healthcare team [20], and with that comes their great responsibility in providing accurate patient education and health information. In our study, most participants endorsed the role of pharmacists in correcting false information about the COVID-19 pandemic in addition to their commitment to self-protection measures discussed previously, which makes an exemplary action for the public to follow. Several independent studies endorsed pharmacists’ perception of their willingness and preparedness in fighting the COVID-19 pandemic [12,13]. Goff, Debra A. et al. discussed international efforts of pharmacists in nine countries by providing crucial frontline care to COVID-19 patients in different settings, including: hospitals, community pharmacies, clinics, long-term care, physician offices, nursing homes, and national and public health [21].

Our study identified specific knowledge gaps related to COVID-19 among pharmacists, as it seems like the younger aged holding bachelor degrees had uncertainties about COVID-19 pandemic having similarities to the Spanish influenza of 1918. Some perceptions in our study revealed gender variations, particularly male pharmacists believed more that they had a role in decreasing COVID-19 spread, while female pharmacists believed they knew more about the difference between influenza and the COVID-19 pandemic. Several studies tried to establish relationship between an influenza vaccine and COVID-19, especially the concept of trained immunity [22,23]. In our study, pharmacists expressed expectations about the ability of an influenza vaccine to decrease the severity and spread of COVID-19. Just like our study, several studies attributed part of the distorted knowledge of pharmacists to considering media as their source of information about the pandemic [13,24]. The diminished confidence of pharmacists toward their ability to swab test for COVID-19 infection is particularly explained by the current non-supportive regulations in Jordan that prohibit involvement of pharmacists in testing for COVID-19 infection, in addition to regulations which prohibit pharmacists from administering COVID-19 vaccines. Fortunately, our study findings could create opportunities for improvement toward targeting public health literacy of pharmacists considering their crucial roles as health educators.

Pharmacists expressed high expectation of a second COVID-19 wave, but as we dug into our findings, pharmacists with higher education degrees (master’s and doctoral) believed more that the second wave will be more severe and the second wave was to be expected within three months by the time they filled out our survey. Nevertheless, pharmacists in our study believed more that the coronavirus vaccine will be effective in COVID-19 prevention. Our findings are consistent, in terms of positive intentions, with a study among French healthcare workers, including pharmacists who were motivated to get the novel COVID-19 vaccine [25].

Pharmacists’ role as professional frontline healthcare members must be supported considering the accumulated evidence of pharmacist’s role as health educators, and interprofessional communicators for the better health of patients and sustainability of health care system, especially during crises and pandemics, such as COVID-19.

Limitations of this study include the involvement of an online survey instead of in-person encounters, causing a small number of respondents due to poor response rates; the reliability and authenticity of the study data might be affected since it was obtained through social network platforms (Facebook and WhatsApp). Nevertheless, these social network platforms are considered official ways of communication in Jordan. Considering the physical and social distancing measures during the current COVID-19 pandemic, using such methodology was the best possible approach. Other alternative and more authentic approaches could have been used (like professional emails) but we had concerns of reduced engagement with participants as they might not frequently check their emails. Data representation of our study population might pose a limitation, since most participants are from the capital of Jordan, Amman, where most healthcare facilities are located.

## 5. Conclusions

Pharmacists are part of the healthcare team and have a great responsibility toward public health during crises and pandemics such as COVID-19. By their exemplary actions, pharmacists in Jordan are committed in using protection measures to minimize the spread of COVID-19 to self and others. Health education and public health awareness are both crucial positive roles of pharmacists, and so comes great responsibility upon pharmacists to stay updated in order to provide evidence-based information to the public and other professionals. Media streaming of health-related information should not be taken lightly, and future measures are recommended to ensure their validity and credibility, which is directly reflected on public and professional common knowledge of our health and diseases.

## Figures and Tables

**Table 1:** Socio-demographic details of pharmacists who responded to the study questionnaire, (*N* = 311).

Parameter	*N* (%)
Age in years	
23–30	140 (45.0%)
31–40	103 (33.1%)
41–50	49 (15.8%)
>50	19 (6.1%)
Gender	
Female	258 (83.0%)
Male	53 (17.0%)
Education level	
Bachelor’s degree	240 (77.2%)
Master’s degree	55 (17.7%)
Ph.D degree	16 (5.1%)
Residence	
Capital City (Amman)	119 (38.3%)
Middle area of Jordan (Al-Zarqa, Al-Balqa, Madaba)	109 (35.0%)
North area of Jordan (Irbid, Al-Mafraq, Ajloun, Jerash)	46 (14.8%)
South area of Jordan (Al-Aqaba, Maan, Al-Tafilah, Al-Karak)	37 (11.9%)
Source of information about the COVID-19 pandemic	
Social networking	121 (38.9%)
TV newscast	74 (23.8%)
Specialized scientific journals	58 (18.6%)
Personal readings	48 (15.4%)
Other sources	10 (3.2%)
Pharmacist workplace	
Community pharmacy	123 (39.5%)
Hospital pharmacy	18 (5.8%)
Others ^a^	168 (54.0%)

^a^: other disciplines of pharmacy profession in Jordan include, but not limited to, academia, registration, quality, marketing, and insurance.

**Table 2:** Pharmacists’ knowledge, attitudes, and practices during the COVID-19 pandemic, (*N* = 311).

Parameter	*N* (%)
Pharmacist is committed to social distancing	
Yes	269 (86.5%)
Sometimes	34 (10.9%)
No	4 (1.3%)
Pharmacist is committed to wearing a mask	
Yes	237 (76.2%)
Sometimes	52 (16.7%)
No	20 (6.4%)
Pharmacist is committed to wearing gloves	
Yes	183 (58.8%)
Sometimes	90 (28.9%)
No	38 (12.2%)
Pharmacist has a role in decreasing COVID-19 spread	
Yes	152 (48.9%)
No	18 (5.8%)
Maybe	140 (45.0%)
Pharmacist knows the difference between influenza and the COVID-19 pandemic	
Yes	263 (84.6%)
No	14 (4.5%)
Maybe	33 (10.6%)
Pharmacist knows any similarities between the COVID-19 pandemic and the Spanish influenza of 1918	
Yes	76 (24.4%)
No	39 (12.5%)
Maybe	104 (33.4%)
Never heard about the Spanish influenza of 1918	91 (29.3%)
Pharmacist corrects the false information about the COVID-19 pandemic	
Yes	237 (76.2%)
No	17 (5.5%)
Maybe	57 (18.3%)
Pharmacist is capable of taking samples to test for coronavirus	
Yes	67 (21.5%)
No	170 (54.7%)
Maybe	74 (23.8%)

**Table 3:** Pharmacists’ expectations about current and future COVID-19 pandemics, (*N* = 311).

Parameter	*N* (%)
Expectation of a second COVID-19 wave	
Yes	151 (48.6%)
No	53 (17.0%)
Maybe	105 (33.8%)
Expectation of the severity of the second COVID-19 wave	
Less severe compared with the first COVID-19 wave	109 (35.0%)
More severe compared with the first COVID-19 wave	135 (43.4%)
No relation between the severity of first and second waves	38 (12.2%)
Same severity as the first COVID-19 wave	27 (8.7%)
Expected time of the second COVID-19 wave?	
Within 3 months	182 (58.5%)
Within one year	72 (23.2%)
Within 2 years	10 (3.2%)
More than 2 years	40 (12.9%)
Expectation of the ability of an influenza vaccine to decrease the severity and spread of COVID-19	
Yes	82 (26.4%)
No	134 (43.1%)
Maybe	95 (30.5%)
Expectation of the presence of a COVID-19 vaccine within 6 months	
Yes	173 (55.6%)
No	46 (14.8%)
Maybe	91 (29.3%)
Expectation about the effectiveness of a COVID-19 vaccine	
Yes	189 (60.8%)
No	22 (7.1%)
Maybe	100 (32.2%)

**Table 4:** Association between pharmacists’ characteristics and their knowledge, attitudes, practices, and expectations towards the COVID-19 pandemic, (*N* = 311).

Parameter	Age	Gender	Residence	Education Level	Workplace
	Chi-square *p*-Value *				
Pharmacist is committed to social distancing					
*p* value	0.596	0.886	0.25	0.352	0.193
df	6	2	6	4	4
X^2^ cutoff value	4.597	0.242	7.801	4.418	6.089
Pharmacist is committed to wearing a mask					
*p* value	0.882	0.489	0.211	0.927	0.420
df	6	2	6	4	4
X^2^ cutoff value	2.377	1.431	8.391	0.880	3.898
Pharmacist is committed to wearing gloves					
*p* value	0.719	0.732	0.080	0.565	0.013 *
df	6	2	6	4	4
X^2^ cutoff value	3.689	0.624	11.289	2.958	12.586
Pharmacist has a role in decreasing COVID-19 spread					
*p* value	0.687	0.010 *	0.008 *	0.571	0.048 *
df	6	2	6	4	4
X^2^ cutoff value	3.926	9.132	17.417	2.921	9.568
Pharmacist knows the difference between the flu and COVID-19 pandemic					
*p* value	0.684	0.032 *	0.273	0.533	0.051
df	6	2	6	4	4
X^2^ cutoff value	3.949	6.878	7.554	3.151	9.460
Pharmacist knows of any similarities between the COVID-19 pandemic and the Spanish influenza of 1918					
*p* value	<0.001 *	0.103	0.337	0.018 *	0.144
df	9	3	9	6	6
X^2^ cutoff value	29.852	6.186	10.167	15.327	9.571
Pharmacist corrects the false information about the COVID-19 pandemic					
*p* value	0.961	0.324	0.173	0.050 *	0.599
df	6	2	6	4	4
X^2^ cutoff value	3.897	2.251	9.013	9.477	2.758
Expectation of a second COVID-19 wave					
*p* value	0.118	0.567	0.017 *	0.07 *	0.559
df	6	2	6	4	4
X^2^ cutoff value	10.158	1.136	15.510	8.651	2.995
Expectation of the severity of the second COVID-19 wave					
*p* value	0.282	0.494	0.108	<0.001 *	0.832
df	9	3	9	6	6
X^2^ cutoff value	10.908	2.399	14.438	30.665	2.816
Expected time of the second COVID-19 wave					
*p* value	0.349	0.624	0.151	0.016 *	0.188
df	9	3	9	6	6
X^2^ cutoff value	10.020	1.758	13.256	15.552	8.756
Expectation of ability of a flu vaccine to decrease the severity and spread of COVID-19					
*p* value	0.084	0.216	<0.001 *	0.131	0.064
df	6	2	6	4	4
X^2^ cutoff value	11.146	3.061	24.395	7.101	8.895
Expectation about the effectiveness of a COVID-19 vaccine					
*p* value	<0.001 *	0.132	0.339	0.819	0.662
df	6	2	6	4	4
X^2^ cutoff value	26.238	4.054	6.803	1.545	2.403

Chi-square test was used; df: degree of freedom; *: indicates statistical significance (*p*-value < 0.05).

## Data Availability

The data presented in this study are available on request from the corresponding author. The data are not publicly available due to privacy concerns.
